# Current Advances in Patient-Perceived Quality Assessment Within Romanian Healthcare

**DOI:** 10.7759/cureus.70111

**Published:** 2024-09-24

**Authors:** Karoly Bancsik, Madalina Diana Daina, Timea Claudia Ghitea, Raluca Bancsik, Lucia Georgeta Daina

**Affiliations:** 1 Trauma, University of Oradea, Oradea, ROU; 2 Public Health, University of Oradea, Oradea, ROU; 3 Pharmacy, University of Oradea, Oradea, ROU; 4 Emergency Medicine, Clinical Emergency Hospital "Avram Iancu", Oradea, ROU; 5 Psycho-Neurosciences and Recovery, University of Oradea, Oradea, ROU

**Keywords:** quality assessment, healthcare administration, servqual, leiden perioperative care patient satisfaction questionnaire (lppsq), patient-centered care healthcare disparities

## Abstract

The global healthcare landscape is shifting toward patient-centered care, emphasizing the integration of patient feedback into service delivery. Romania, aligning with this trend, has implemented patient-perceived quality assessment tools to enhance healthcare services and better meet patient needs and expectations. This study aims to review comprehensively the implementation and impact of these tools in Romania, focusing on their role in improving healthcare quality. By examining key assessment instruments such as the Patient Satisfaction Questionnaire (PSQ), the Service Quality (SERVQUAL) model, and the Romanian Healthcare Quality Assessment Survey (RHQAS), the research seeks to understand how these tools have been used to identify areas for improvement and drive advancements in patient care.

Employing a comprehensive review methodology, the study will conduct a thorough literature search to identify relevant studies, reports, and publications, analyzing the PSQ, SERVQUAL, and RHQAS in detail to understand their measurement domains, psychometric properties, and application within Romania. Additionally, qualitative data from interviews with healthcare providers and patients may be collected to offer further insights into the use and effectiveness of these tools. The study's findings are expected to provide valuable insights into the role of patient-perceived quality assessment tools in enhancing healthcare in Romania, identifying strengths, weaknesses, and opportunities for improvement. The results will highlight the effectiveness of combining international methodologies with localized adaptations to address the specific needs of the Romanian healthcare system, ultimately contributing to the ongoing efforts to improve patient satisfaction and health outcomes by informing the development and refinement of patient-centered care initiatives in Romania.

## Introduction and background

In recent years, there has been a growing emphasis on patient-centered care, which prioritizes the needs, preferences, and values of patients [1]. Consequently, healthcare professionals and policymakers are increasingly dependent on patient-perceived quality evaluation methods to evaluate the quality of care from the patient's point of view. These tools are crucial for boosting healthcare services, optimizing patient pleasure, and guaranteeing accountability [2-4].

The concept of patient-perceived quality is not a modern phenomenon. Historical records indicate that ancient civilizations recognized the importance of patient feedback in healthcare [5]. In ancient Greece, the Hippocratic Oath emphasized ethical conduct and the need for physicians to treat patients with respect and compassion [6,7]. Similarly, ancient Chinese medicine, as documented in texts such as the Huangdi Neijing, placed significant importance on understanding patient experiences and perceptions to guide treatment [8].

The nineteenth century marked significant advancements in medical practice, leading to the formalization of patient care. During this period, Florence Nightingale, a pioneering figure in nursing, introduced systematic methods for assessing patient experiences and outcomes. Nightingale’s work during the Crimean War highlighted the importance of sanitary conditions and compassionate care in improving patient recovery. Her efforts laid the groundwork for future quality assessment in healthcare, emphasizing the role of patient comfort and satisfaction in medical outcomes [9-11].

The early twentieth century saw the development of more structured approaches to assessing patient satisfaction. Hospitals and healthcare providers began to recognize the value of patient feedback in improving care delivery. The concept of "patient satisfaction" started to gain traction, with hospitals conducting informal surveys and interviews to gather patient opinions. These early efforts were often ad hoc and lacked standardized methodologies, but they marked the beginning of a more formalized approach to understanding patient experiences [4,12-14].

The period following World War II witnessed significant advancements in medical technology and healthcare delivery, leading to increased attention to patient experiences [15,16]. In the 1950s, American humanistic psychologist Carl R. Rogers developed the concept of client-centered therapy, emphasizing the importance of understanding and addressing the individual's experiences and emotions. This approach was subsequently introduced into the medical field by psychoanalyst Michael Balint, who coined the term "patient-centered medicine". Balint's work highlighted the significance of the physician-patient relationship and the need for medical practitioners to consider the patient's psychological and emotional state alongside their physical health [17-21].

The 1970s and 1980s were pivotal decades for the field of patient-perceived quality assessment. During this period, researchers developed several standardized tools for measuring patient satisfaction. One of the most notable contributions was the development of the Patient Satisfaction Questionnaire (PSQ) by Ware et al. in 1983. The PSQ provided a comprehensive framework for assessing patient satisfaction across multiple dimensions, including technical quality, interpersonal aspects, and accessibility [22-24].

Another significant development was the introduction of the Service Quality (SERVQUAL) model by Parasuraman et al. in 1988. Originally developed for the service industry, SERVQUAL was adapted for healthcare to measure the gap between patient expectations and perceptions of service delivery. The model focused on five key dimensions: tangibles, reliability, responsiveness, assurance, and empathy, providing a detailed analysis of service quality gaps [25-27].

The 1990s marked a shift toward patient-centered care, a paradigm that placed patients at the center of healthcare delivery. This period saw the development of tools that not only measured patient satisfaction but also assessed patient experiences and engagement. The Picker Institute, founded in 1994, played a crucial role in promoting patient-centered care by developing patient experience surveys and advocating for the integration of patient perspectives into healthcare decision-making [28,29]. The Institute of Medicine (IOM) also published influential reports, such as "Crossing the Quality Chasm" in 2001, which emphasized the importance of patient-centered care and the need for healthcare systems to prioritize patient experiences [30].

The early 2000s saw the implementation of national and international initiatives to standardize patient-perceived quality assessment. In the United States, the Centers for Medicare and Medicaid Services (CMS) introduced the Hospital Consumer Assessment of Healthcare Providers and Systems (HCAHPS) survey in 2006. HCAHPS provided a standardized tool for measuring patients' perspectives on hospital care, allowing for national comparisons and benchmarking. The survey covered various domains, including communication with nurses and doctors, pain management, and discharge information, providing a comprehensive assessment of patient experiences [5,31-35].

Internationally, countries such as the United Kingdom (UK), Canada, and Australia developed their own patient experience surveys to assess healthcare quality. The National Health Service (NHS) in the UK, for example, introduced the National Patient Survey Programme, which included standardized surveys to gather patient feedback on various aspects of care. These initiatives highlighted the global recognition of the importance of patient-perceived quality and the need for standardized assessment tools [36,37].

The 2010s witnessed significant technological advancements that transformed the landscape of patient-perceived quality assessment. The widespread adoption of electronic health records (EHRs) and patient portals enabled real-time feedback and continuous monitoring of patient perceptions [38,39]. Studies demonstrated the effectiveness of patient portals in enhancing patient engagement and satisfaction by providing timely access to health information and communication with healthcare providers [40-42].

Mobile health applications and telemedicine platforms also emerged as valuable tools for collecting patient feedback. Telemedicine significantly improved patient satisfaction, particularly in remote and underserved areas, by offering convenient and accessible healthcare services. In recent years, the integration of advanced analytics, artificial intelligence (AI), and machine learning has enabled the analysis of large datasets that allow healthcare providers to make data-driven decisions. These technological advancements provided new avenues for more dynamic and responsive quality assessment methods [43-46].

## Review

Current quality assessment tools used worldwide

Across the world, a diverse range of tools is employed to ensure that healthcare systems align with patient needs and expectations. From widely recognized instruments like the HCAHPS in the United States to region-specific tools like the European Task Force on Patient Evaluations of General Practice Care (EUROPEP) questionnaire in Europe, these assessment methods help drive improvements in healthcare delivery, promote transparency, and support informed decision-making by patients and healthcare providers alike.

HCAHPS

The HCAHPS is a standardized survey used in the United States to measure patients' perceptions of their hospital experiences. Developed by the CMS in partnership with the Agency for Healthcare Research and Quality (AHRQ), the survey aims to provide a national standard for collecting and publicly reporting patients' perspectives on hospital care. The HCAHPS survey covers various aspects of the patient experience, including communication with nurses and doctors, the responsiveness of hospital staff, cleanliness and quietness of the hospital environment, pain management, communication about medicines, and discharge information. The results of the survey are used to compare hospitals, improve the quality of care, and provide transparency to the public, helping patients make informed decisions about where to receive care. The HCAHPS survey plays a critical role in assessing patient-perceived quality in hospitals, offering numerous advantages such as standardization, transparency, and actionable feedback [5,34,47-49].

Patient Experience Questionnaire (PEQ)

The PEQ has a significant impact on healthcare services by providing actionable feedback for healthcare providers to improve the quality of care. The detailed patient feedback helps healthcare providers pinpoint specific areas needing improvement, allowing for targeted quality improvement initiatives. The PEQ also promotes patient-centered care by ensuring that patient voices are heard and valued, enhancing patient satisfaction and loyalty. The regular use of the PEQ enables continuous monitoring of performance trends over time, helping to assess the impact of implemented changes and informing policy decisions and quality improvement initiatives. The PEQ is designed to be adaptable to different healthcare settings, such as hospitals, clinics, and general practices. By systematically collecting this feedback, healthcare organizations can identify strengths and areas for improvement, ultimately leading to enhanced patient-centered care and better health outcomes. The data from PEQs can be used for benchmarking and comparing patient experiences across different providers or regions, contributing to broader efforts to improve healthcare quality [50-52].

Patient-Reported Outcomes Measurement Information System (PROMIS)

The PROMIS is an initiative by the National Institutes of Health (NIH) designed to improve the measurement of patient-reported outcomes (PROs) in clinical research and practice. PROMIS tools assess multiple health domains such as physical health, mental health, and social well-being [53,54]. The system uses item response theory (IRT) to create dynamic, adaptive surveys that are precise and efficient, allowing for tailored assessments based on individual patient responses. The PROMIS offers numerous advantages, including comprehensive and flexible assessments, precision and efficiency, standardization, patient-centeredness, and versatility. By focusing on patient-reported outcomes, the PROMIS helps to ensure that the patient’s voice is central in evaluating the effectiveness of treatments and interventions, guiding clinical decision-making, and improving overall healthcare quality [50,55,56].

SERVQUAL

The SERVQUAL model is a widely used framework for assessing service quality by measuring the gap between customer expectations and their perceptions of the actual service received. The model evaluates service quality across five key dimensions: tangibles (the physical appearance of facilities and equipment), reliability (the ability to perform the promised service dependably), responsiveness (the willingness to help customers and provide prompt service), assurance (the knowledge and courtesy of employees and their ability to inspire trust), and empathy (the provision of caring and individualized attention to customers). By analyzing these dimensions, SERVQUAL helps organizations identify areas where service delivery can be improved to better meet customer expectations. The SERVQUAL model offers advantages such as identifying specific service gaps, providing customer-centric insights, and being adaptable across various industries. The model also delivers actionable feedback for targeted improvements and supports benchmarking and enhanced communication within organizations to drive continuous improvement in service delivery [25,57,58].

Press Ganey Survey (PGS)

The PGS is widely used in the United States to measure patient satisfaction across various healthcare settings, including hospitals, outpatient services, and long-term care facilities. The PGS collects data on several aspects of patient care, including the likelihood to recommend the hospital, the overall rating of care, and specific aspects such as wait times, communication with healthcare providers, and facility cleanliness. The PGS offers several advantages, including comprehensive data collection, benchmarking, actionable insights, patient-centered care, and reputation enhancement [59-63].

Other Tools

Other notable tools include the Picker Patient Experience Questionnaire (PPE-15), the Consumer Assessment of Healthcare Providers and Systems (CAHPS) for specific settings like nursing homes and clinician and group practices, and the Net Promoter Score (NPS), which is used to gauge overall patient loyalty and satisfaction.

The PPE-15 emphasizes patient-centered care by evaluating dimensions such as communication, involvement in care decisions, respect for patient preferences, emotional support, coordination of care, and the physical comfort of the healthcare environment. The questionnaire is widely used to gather feedback directly from patients, helping healthcare providers identify areas for improvement and enhance the overall quality of care from the patient's perspective. The questionnaire offers several advantages, including its focus on patient-centered care, evidence-based development, ease of use, actionable insights, and versatility [64,65].

The CAHPS surveys are tailored to different healthcare settings, such as hospitals, outpatient clinics, nursing homes, and health plans, allowing for a broad assessment of patient experiences across the healthcare continuum. The data collected through these surveys is used by healthcare providers, policymakers, and consumers to evaluate and compare the quality of care provided by different organizations. This helps to drive improvements in healthcare services and supports informed decision-making by patients when choosing healthcare providers. One of the key strengths of the CAHPS program is its focus on the patient perspective, ensuring that the feedback collected is directly relevant to patients' needs and expectations. The results of the CAHPS surveys are often publicly reported, contributing to transparency and accountability in healthcare, and helping to promote a patient-centered approach to care [66-68].

The NPS is a widely recognized metric used to measure customer loyalty and satisfaction by assessing how likely customers are to recommend a product, service, or company to others. The NPS is determined by asking customers a single key question. The NPS is valued for its simplicity and effectiveness in providing a clear, actionable measure of customer sentiment. Organizations use the NPS to identify areas for improvement, monitor customer loyalty over time, and benchmark their performance against industry standards. In healthcare, the NPS is particularly useful for understanding patient satisfaction and informing strategies to enhance patient care and experience. The NPS offers several advantages, including simplicity, actionable insights, benchmarking capability, focus on loyalty, and strategic insights [69-71].

Current quality assessment tools used in Romania

In Romania, various types of questionnaires are utilized to assess patient satisfaction and perceived quality in healthcare settings. These questionnaires are designed to capture multiple dimensions of patient experience, including communication with healthcare providers, the efficiency of service delivery, and the overall quality of care received. Some of the commonly used questionnaires include the PSQ, the SERVQUAL model, HCAHPS, the EUROPEP questionnaire, the Romanian Healthcare Quality Assessment Survey (RHQAS), the WHO Quality of Life (WHOQOL) questionnaire, and customized institutional surveys.

PSQ

The PSQ is a widely used tool designed to measure patients' perceptions of the quality of healthcare services they receive. Developed by Ware et al. in 1983, the PSQ is one of the most comprehensive instruments for assessing various dimensions of patient satisfaction. It provides valuable insights into patients' experiences and identifies areas for improvement in healthcare delivery [22,23].

The PSQ is utilized globally, including in Romania, where it plays a crucial role in assessing healthcare quality. By capturing detailed feedback on multiple aspects of patient care, the PSQ supports healthcare providers in their efforts to enhance service quality and patient satisfaction [72].

EUROPEP Questionnaire

The EUROPEP questionnaire is a widely used tool designed to measure patient experiences and satisfaction with primary care services in Europe. Developed by the European Task Force on Patient Evaluations of General Practice Care, the EUROPEP questionnaire provides a comprehensive assessment of patients' perspectives on the quality of care they receive from general practitioners. It aims to capture key dimensions of patient care that are crucial for continuous improvement in primary healthcare settings [73,74].

The EUROPEP questionnaire typically employs a Likert scale for responses, where patients rate their experiences on a scale ranging from very poor to excellent. This standardized rating system allows for easy quantification and analysis of patient feedback. The questionnaire is usually administered through mail, online surveys, or in-person visits to healthcare facilities, ensuring broad accessibility for patients [73,75].

RHQAS

The RHQAS is designed to evaluate patient experiences and satisfaction within the Romanian healthcare system. It aims to provide a comprehensive assessment of various aspects of healthcare delivery, focusing on the quality of services, patient safety, and overall patient satisfaction. The survey is a valuable tool for assessing patient satisfaction and perceived quality of care within the Romanian healthcare system. Its comprehensive, patient-centered approach provides detailed insights into various aspects of healthcare delivery, facilitating continuous improvement and informed policy development [76,77].

WHOQOL

The WHOQOL questionnaire is a comprehensive tool developed by the WHO to assess individuals' perceptions of their quality of life. Unlike many other health assessment tools that focus solely on physical health, the WHOQOL questionnaire encompasses a broad range of factors, including psychological, social, and environmental dimensions. It is designed to be applicable across different cultures and settings, providing a universal measure of the quality of life [77,78].

The WHOQOL questionnaire was developed in response to the need for a standardized tool to measure the quality of life in a way that is relevant and applicable worldwide. The development process involved extensive international collaboration, including input from diverse cultural and socio-economic backgrounds. The primary purpose of the WHOQOL is to provide a comprehensive, multi-dimensional assessment of the quality of life that can be used in clinical practice, research, and policy-making [79,80].

Customized Institutional Surveys

Customized institutional surveys are tailored tools developed by healthcare institutions to assess specific aspects of patient experiences and satisfaction unique to their settings. These surveys are designed to address particular needs, goals, and contexts of the institution, providing detailed and actionable feedback that can be used to improve healthcare delivery and patient outcomes. Unlike standardized surveys, customized surveys allow institutions to focus on areas most relevant to their patient population and operational strategies [4,36,77].

Key features of assessment tools

Patient-perceived quality assessment tools are essential for evaluating healthcare services from the patient's perspective, providing valuable insights into the effectiveness, safety, and satisfaction associated with healthcare delivery. This paper reviews several prominent tools, including the HCAHPS, PROMIS, PGS, PPE-15, CAHPS, NPS, PSQ, EUROPEP questionnaire, RHQAS, WHOQOL questionnaire, and customized institutional surveys. Each tool, designed with specific objectives, offers unique features that contribute to a comprehensive understanding of patient experiences. Key features of these assessment tools include standardization, which ensures consistency in data collection and enables comparisons across institutions and time periods [4,81]. A brief presentation of the key features is presented in Table 1.

**Table 1:** The key features of assessment tools HCAHPS: Hospital Consumer Assessment of Healthcare Providers and Systems; PEQ: Patient Experience Questionnaire; PROMIS: Patient-Reported Outcomes Measurement Information System; SERVQUAL: Service Quality; PGS: Patient Satisfaction Survey; PPE-15: Picker Patient Experience Questionnaire-15; CAHPS: Consumer Assessment of Healthcare Providers and Systems; NPS: Net Promoter Score; PSQ: Patient Satisfaction Questionnaire; EUROPEP: European Task Force on Patient Evaluations of General Practice; RHQAS: Romanian Healthcare Quality Assessment Survey; WHOQOL: World Health Organization Quality of Life; WHOQOL-100: 100-item WHOQOL; WHOQOL-BREF: WHOQOL Brief Version

Current assessment tools	Key features
HCAHPS [47,82-84]	It provides a national standard. Standardized questions ensure consistency in data collection. Results are publicly reported. Patients can use HCAHPS data to make informed decisions about where to receive care. The survey covers a wide range of domains, including communication, pain management, and discharge information. It focuses on aspects of care that are important to patients. It provides healthcare providers with actionable feedback to identify areas for improvement and implement targeted interventions. Data from HCAHPS can inform policy decisions and quality improvement initiatives at both the hospital and national levels.
PEQ [50,51,85,86]	It covers multiple domains of patient care, including communication with healthcare providers, the responsiveness of hospital staff, pain management, and discharge information. It addresses various dimensions of patient care, such as emotional support, respect for patient preferences, coordination of care, and involvement of family and friends. It is designed to capture the patient's perspective. By focusing on aspects of care that matter most to patients, the PEQ can help healthcare providers improve patient satisfaction and loyalty. It is relatively short and straightforward, making it easy for patients to complete. The brevity of the questionnaire allows for quick administration and analysis. It provides healthcare providers with specific, actionable feedback on key aspects of patient care. It can be used across different healthcare settings, including hospitals, outpatient clinics, and long-term care facilities. The standardized format allows for comparison across different departments and institutions.
PROMIS [50,55,56,87]	It assesses a wide range of health domains including physical, mental, and social health. It makes use of the item response theory (IRT). Computerized adaptive testing (CAT) selects the most relevant questions for each patient. It has adaptive testing and precise measurement. It provides standardized measures. The standardized approach ensures consistent data collection and interpretation. It focuses on outcomes that matter to patients. It empowers patients to actively participate in their care and health assessment. It can be used in clinical research, routine clinical practice, and population health studies. It can be integrated into electronic health records.
SERVQUAL [25,57,58]	It can be used across different service industries, including healthcare, making it a versatile tool for evaluating service quality. There are five dimensions: tangibility, reliability, responsiveness, empathy, and assurance. It identifies gaps between customer expectations and perceptions of the actual service received. It provides actionable insights for targeted quality improvement initiatives. It provides a dual focus on expectations and perceptions. It helps identify both strengths and weaknesses in service delivery, enabling organizations to build on their strengths and address their weaknesses. It provides quantitative data that can be analyzed to measure service quality over time, track performance, and assess the impact of quality improvement initiatives. The use of structured questionnaires helps in obtaining objective and comparable data.
PGS [59-62]	It collects detailed feedback on various aspects of patient care, including communication, wait times, facility cleanliness, and overall satisfaction. It is adaptable to various healthcare settings, including hospitals, outpatient services, and long-term care facilities. It provides hospitals with the ability to benchmark their performance against national and regional standards. The standardized format allows for comparative analysis across different departments within a hospital or across multiple facilities. The detailed patient feedback helps healthcare providers pinpoint specific areas needing improvement. The regular use of PGS enables continuous monitoring of performance trends over time. By focusing on patient feedback, the PGS encourages a patient-centered approach to healthcare. Addressing feedback from PGS can lead to enhanced patient satisfaction and loyalty. The obtained data analytics can help healthcare organizations to better understand their patients and their needs. Sharing PGS results with the public fosters transparency and builds trust between healthcare providers and the community.
PPE-15 [64,65,88,89]	It was developed based on extensive research, including literature reviews, patient interviews, and pilot testing. The questionnaire has been validated in multiple studies. It is concise and easy to administer. The brevity of the questionnaire allows for quick administration and analysis. The insights gained from the PPE-15 can inform targeted quality improvement initiatives. It can be used across various healthcare settings, including hospitals, outpatient clinics, and long-term care facilities. It allows for comparison across different departments and institutions.
CAHPS [66-68,90]	It provides a national standard for measuring and comparing patient experiences across different healthcare providers and settings. Standardized questions ensure consistency in data collection. The surveys cover a wide range of domains, including communication with healthcare providers, access to care, and patient experiences with healthcare services. There are specific CAHPS surveys for different healthcare settings, such as hospitals, nursing homes, and outpatient services. Results are often publicly reported. Patients can use CAHPS data to make informed decisions about their healthcare providers. The surveys prioritize the patient's perspective. By systematically capturing patient feedback, CAHPS empowers patients to participate actively in their healthcare evaluation. Detailed feedback from CAHPS surveys provides healthcare providers with specific, actionable insights to identify areas for improvement. The regular administration of CAHPS surveys allows for continuous monitoring of performance trends over time.
NPS [70,71,91]	Its simplicity makes it easy for patients to understand and respond quickly. The brevity of the survey ensures minimal burden on patients. It provides a clear and easily interpretable metric. It is a widely used metric across various industries, enabling healthcare providers to benchmark their performance against other organizations. The standard format allows for consistent data collection and comparability across different departments and facilities. It focuses on the likelihood of recommendation, which is a strong indicator of patient loyalty and future utilization of healthcare services. High NPS scores can correlate with increased word-of-mouth referrals. Regular NPS tracking helps healthcare organizations monitor performance trends over time and assess the impact of quality improvement initiatives. Feedback from NPS can highlight areas where patient experience can be enhanced, driving patient-centered improvements in care delivery.
PSQ [22,23,91]	It covers a wide range of domains, including accessibility and convenience, technical quality, interpersonal manner, communication, financial aspects, and time spent with the doctor. It provides detailed feedback on various aspects of patient care. It is designed to capture the patient's perspective. It uses a structured format with standardized questions. The questions are clear and straightforward. It provides healthcare providers with specific, actionable feedback on key aspects of patient care. It can inform targeted quality improvement initiatives. It provides a standardized measure of patient satisfaction. It enables continuous monitoring of performance trends over time.
EUROPEP [73,92]	It covers a wide range of domains relevant to primary care, including accessibility, continuity, communication, medical care, and organization of services. It provides a holistic view of patient care, addressing both clinical and non-clinical aspects of the healthcare experience. It ensures that experiences and satisfaction are central to the evaluation of primary care services. It can help healthcare providers improve patient satisfaction and loyalty. It is widely used across Europe, providing a standardized measure that allows for cross-country comparisons and benchmarking within the European context. The standardized format ensures consistency in data collection and analysis. It provides healthcare providers with specific, actionable feedback on various aspects of patient care. It can inform targeted quality improvement initiatives. It uses a structured questionnaire with standardized questions, making it easy to administer and analyze. The questions are designed to be clear and understandable for patients.
RHQAS [76,78,93]	It is tailored to the specific context of the Romanian healthcare system, ensuring that it addresses local healthcare issues and patient concerns effectively. It covers a wide range of domains, including accessibility, communication, clinical care, hospital environment, and administrative services. It provides detailed feedback on various aspects of patient care. It is designed to capture the patient's perspective. It provides a standardized measure of patient satisfaction within Romania. The standardized format ensures consistency in data collection and analysis. Insights gained from RHQAS can inform targeted quality improvement initiatives. Data from RHQAS can inform healthcare policy decisions at both the institutional and national levels.
WHOQOL [80,81,94]	It covers multiple dimensions of quality of life, including physical health, psychological health, social relationships, and environment. It provides detailed feedback on various aspects of quality of life. It is a globally recognized tool, making it suitable for cross-cultural comparisons and international research. The instrument has been adapted and validated in multiple languages and cultural contexts. It captures the individual's perspective on their own quality of life. It can help healthcare providers improve overall quality of life and well-being. It provides a standardized measure of quality of life. Its standardized format facilitates benchmarking against national and international standards. Insights gained from WHOQOL can inform targeted interventions aimed at enhancing overall well-being and addressing specific needs. It can be used in various settings, including clinical practice, research, and public health. It is available in several versions, such as WHOQOL-100 and WHOQOL-BREF.
Customized institutional surveys [4,95,96]	They can be designed to address the specific needs and priorities of institutions. Institutions can develop questions that are highly relevant to their specific context. Customized surveys can be easily adapted to changing needs and priorities. Institutions can focus on particular areas of interest or concern, such as specific departments, services, or patient demographics. Customized surveys can be designed to be concise and focused. The detailed and context-specific nature of customized surveys provides institutions with specific, actionable feedback that can be directly applied to improve services and outcomes. The results of customized surveys are immediately relevant to an institution's current context, facilitating timely and effective interventions. Customized surveys can involve various stakeholders in the design process.

## Conclusions

The emphasis on patient-centered care in Romania is well-reflected through the adoption and adaptation of various patient-perceived quality assessment tools within its healthcare system. These tools, ranging from internationally recognized instruments like the PSQ and the SERVQUAL model to localized surveys such as the RHQAS, have been instrumental in capturing diverse dimensions of patient experience, from clinical interactions to overall service efficiency and quality. The utilization of such tools signifies a critical stride towards aligning healthcare services with the needs and expectations of patients, ensuring that the voices of the patients are not only heard but also acted upon.

In the Romanian context, the strategic integration of both global methodologies and tailored local approaches has enabled a more robust assessment of healthcare quality. This dual approach not only supports healthcare providers in enhancing service delivery but also fosters a culture of continuous improvement and accountability. It is essential for Romania to continue refining these tools to better address local healthcare challenges and to sustain the evolution towards an increasingly patient-centered healthcare system. This commitment to quality assessment and improvement is pivotal for enhancing patient satisfaction and, ultimately, the overall health outcomes in Romania.
